# Effects of Oral Appliance Therapy with a Mouth Shield in Periodontitis Patients Who Snore: A Split-Mouth Randomized Controlled Trial

**DOI:** 10.3390/dj13070292

**Published:** 2025-06-27

**Authors:** Ju-Ying Lin, Emet Schneiderman, Jason Hui, Carlos Parra Carrasquer, William Stenberg, Zohre German, Jason Adam Harvey, Preetam Schramm

**Affiliations:** 1 Department of Periodontology, Texas A&M University College of Dentistry, Dallas, TX 75246, USA; cparra@tamu.edu; 2 Department of Biomedical Sciences, Texas A&M University College of Dentistry, Dallas, TX 75246, USA; stenberg@tamu.edu (W.S.); german@tamu.edu (Z.G.); schramm@tamu.edu (P.S.); 3 Department of Comprehensive Dentistry, Texas A&M University College of Dentistry, Dallas, TX 75246, USA; huidds@gmail.com

**Keywords:** sleep apnea, oral appliance, periodontitis, mouth breathing, microbiology

## Abstract

**Background**: Periodontitis is linked to sleep-disordered breathing (SDB), including snoring, with 50–75% of cases involving mouth breathing (MB). Standard treatment includes scaling and root planing (SRP). Oral appliance therapy (OAT) is used to treat snoring and SDB. OAT plus a mouth shield (OAT+) worn during sleep may reduce MB to enhance periodontal health. This study evaluated whether OAT+, as an adjunct to SRP, improves periodontal health by reducing periodontal pathogens and facilitating upper airway patency. **Methods**: Fourteen participants with mild–moderate periodontitis were randomized to receive SRP on one side of the mouth at baseline (T0). Pocket depth (PD), bleeding on probing (BOP), and plaque index (PI) were recorded, and bacterial DNA from periodontal pockets were analyzed via PCR at baseline (T0) and 12 weeks (T3). At 4 weeks (T1), all participants received a self-titrated myTAP^®^ OA, followed by a mouth shield at 8 weeks (T2). Sleep metrics, including respiratory disturbance index (RDI), were recorded using the NOX T3 at T0–T3. **Results**: BOP and deep PD levels exhibited slight improvements from the baseline for both SRP and non-SRP (OAT+ only) treated sites but did not achieve significance. BOP decreased significantly more from the baseline in the SRP than in the non-SRP group at T3 (*p* = 0.028); *P. gingivalis’* presence declined on both sides (*p* = 0.0135). Other periodontal and bacterial parameters showed no significant differences between or within groups. Snoring (*p* = 0.011), MB (*p* = 0.025), and RDI (*p* = 0.019) significantly decreased with OAT+ at T3. **Conclusions**: In mild–moderate periodontitis patients who snore, OAT+ reduces snoring, MB, and obstructive events, serving as an adjunct to SRP with no negative clinical effects over the short term. The combined therapy yielded similar results to OAT+ alone, likely due to minimization of MB. Its capacity to improve the oral environment is worthy of further investigation.

## 1. Background and Rationale

**Periodontitis** is a chronic inflammatory condition that results in loss of periodontal tissue support, often leading to tooth loss. This condition remains a common burden to public health, with recent studies showing a prevalence of 62% among patients pooled from epidemiology data globally between 2011 and 2020 [1]. The pathogenesis is multifactorial. The primary etiologic factor for developing periodontitis is an imbalance in oral microbiome homeostasis. Such perturbations can be due to alterations of the host environment and/or immune status of the host, thus predisposing a site to disease [2]. Therapeutic strategies for treating periodontitis, whether through mechanical debridement or pharmaceutical management, are well documented for targeting the inflammatory pathway. The current standard periodontal therapy includes non-surgical treatment consisting of scaling and root planing (SRP), in addition to oral hygiene instructions [3,4,5]. While these treatments are effective in early disease, the individual response to the microbiome challenges often varies, and the compliance of the patients tends to be inconsistent. Moderate cases may require adjunctive antimicrobial treatments [6] and/or surgical intervention [7], while severe cases often result in tooth loss [8], requiring dentures or implant therapy. Even with treatment, moderate periodontitis is often progressive, requiring a lifetime of periodontal maintenance therapy. The patient’s compliance with smoking cessation and daily self-performed plaque control is paramount to treatment success [9]. With aging, the deterioration of overall health can include the worsening of systemic conditions such as cardiovascular disease, diabetes mellitus, and rheumatoid arthritis; these are also associated with the progression of periodontitis [10,11]. In recent years, obstructive sleep apnea (OSA) has gained more attention as a potential risk factor associated with periodontal disease [12]. Both disorders share risk factors and similar systemic inflammatory mediators with elevated gingival crevicular fluid interleukins and serum C-reative protein levels [13]. OSA is a chronic sleep-related breathing disorder resulting from partial or complete obstruction of the upper airway, inducing oxygen desaturation and causing sleep disruption [14]. The estimated prevalence of OSA in the United States is the second highest in the world at 33.2% after China [15]. Left untreated, OSA may increase the risk of metabolic and cardiovascular disease, depression, and cognitive impairment [16].

A recent systematic review found that the prevalence of periodontitis in patients with OSA ranges from 17.5% to 96.4% across various studies [17,18]. The presenting dental-related symptoms are loud snoring, mouth breathing, and a dry mouth during sleep. Mouth breathing (MB) is often associated with dry mouth, a reduction in saliva flow, and an increased risk of periodontitis. Particularly during sleep, MB is strongly linked to both worsening periodontal health and the development of periodontitis, with approximately 55% to 75% of chronic mouth breathers experiencing gingivitis or periodontitis [19].

Socransky and colleagues conducted a comprehensive analysis of the oral microbiota, leading to the identification of the “red complex”—*P. gingivalis*, *T. forsythia*, and *T. denticola*—which are strongly linked with each other and flourish in the disease sites [20,21]. The keystone pathogen hypothesis was proposed to explain the shift from a symbiotic microbiota to dysbiosis induced by one keystone pathogen [22]. *P. gingivalis* was shown to alter both the amount and composition of the microorganisms by modulating complement function in the host immune response to enhance its survival [23,24]. A 12-month longitudinal study demonstrated that the levels of *P. gingivalis* and *T. denticola* in subgingival plaque were predictive of which sites would show increased periodontitis severity and progression [25]. A similar result was found when the salivary biomarker alanine aminotransferase (ALT) was combined with *P. gingivalis*; this could serve as a diagnostic tool to predict disease progression [26]. Recently, under the advances in genome sequencing technology, unnamed/uncultured species were discovered and more genera were linked to periodontitis. *Fusobacterium* and *Porphyromonas* were still associated with periodontitis cases, while *Rothia* and *Streptococcus* in the majority of healthy sites [27,28]. Later, Van Dyke et al. proposed that the dysbiotic microbiome found in the gingival sulcus might not be the major initiator but rather the whole oral biofilm; the inflammation created by the host immune response should be viewed as the principle driver for the disease’s development and exacerbator of its progression [29]. The susceptible host/genetic determinants and oral environment also contribute to microbiome dysbiosis [30]. Plaque-retentive factors [31], such as faulty restorations, can promote bacterial accumulation, while conditions like deeper periodontal pockets and xerostomia from medications or from MB create a favorable environment for dysbiotic changes [32]. An observational study found that MB from the late adolescent group increased *S. mutans* levels and higher plaque scores with respect to the control subjects [33]. A drier mouth with reduced salivary flow, reduced self-cleansing, lower pH levels, and OSA-related intermittent hypoxia may possibly lead to changes in the oral microbiome. Recent studies suggest that OSA and MB are associated with bacterial species and genera linked to periodontitis [34]. Patients with severe OSA appear to harbor higher amounts of periodontal pathogens compared with those with mild or moderate OSA or control subjects [35].

Conventionally, the first choice of treatment for OSA is continuous positive airway pressure (CPAP) therapy. However, due to factors such as cost, inconvenience, and patients’ preference, oral appliance therapy (OAT) is now considered a viable alternative for managing OSA. OAT creates an enlarged oropharyngeal space by advancing the mandible and tongue [36]. It decreases the airway’s collapsibility to enable proper breathing. OAT is also recommended as the first choice of treatment for mild to moderate OSA and the second line of therapy for severe OSA [37] or in those who refuse CPAP therapy [38]. OAT paired with a mouth shield (OAT+) that fits over the labial surface and extends into the fornices of the vestibule has been used to improve comfort, address nasal breathing, and reduce oral dryness [39,40].

While the effectiveness of mechanical debridement in managing periodontal disease is well documented, to our knowledge, the potentially additive effects of OAT+ on periodontitis patients with MB have not been investigated. We theorized that optimizing the oral environment by reducing MB could lessen the overgrowth of periodontal pathogens and minimize inflammation and improve periodontal health. We asked the question whether the combined treatment for OSA and periodontitis can yield similar or superior outcomes to OAT+ alone.

Using a split-mouth design (with or without SRP), our primary objective was to test that OAT+ used during sleep would reduce MB and modulate the host immune response with a reduction in OSA events. Additionally, mechanical debridement with SRP on one side of the mouth was expected to be more effective compared with the other side of the mouth receiving OAT+ only. We measured periodontal health outcomes including probing pocket depth (PPD), bleeding on probing (BOP), and changes in oral flora levels after 12 weeks (primary outcome measures). Our secondary objectives were to test whether OAT+ would reduce MB, snoring, the oxygen desaturation index (ODI), and the respiratory disturbance index (RDI) at 12 weeks compared with the baseline (secondary outcome measures).

## 2. Materials and Methods

### 2.1. Study Design

Snoring adults with respiratory disturbance indices (RDI, detailed below) consistent with OSA, and mild to moderate periodontitis diagnoses were enrolled in the study. Participants were evaluated at baseline, prior to any intervention (T0), and at 4 weeks (T1), 8 weeks (T2), and 12 weeks (T3). A split-mouth design was used where each participant was randomly assigned to receive SRP on either the right or left side of the mouth (Right-SRP and Left-SRP groups). The mouth sides were also contrasted according to whether they received OAT+ versus OAT+ and SRP (NSRP and SRP groups). The timetable of the interventions, assessments, and data collection time points is shown in Supplementary File S1.

On the basis of our previous work [36,37], a sample of 27 subjects was targeted to demonstrate medium-sized effects using dependent-sample analyses (i.e., for before–after and split-mouth comparisons) with 80% power and an alpha of 5%.

#### 2.1.1. Participants and Eligibility Criteria

Individuals ≥ 18 years old with mild to moderate periodontitis (generalized periodontitis Stage I–II Grade A–B) according to the 2017 Classification of Periodontal and Peri-Implant Diseases and Conditions [41] were eligible for enrollment. Additional key inclusion criteria included:

− Subjects who had not undergone periodontal treatment in the past 6 months;
− Home sleep test (HST) confirmation of MB, snoring, and a respiratory disturbance index (RDI). More than 5 apnea, hypopnea, respiratory effort-related arousal (RERA) and snoring RERA events/hour of recording time;
− Those who had not used an oral appliance in the past;
− Unobstructed nasal breathing assessed during the oral exam (ability to breathing through the nose for 1–2 min with the mouth closed);
− At least 8 stable teeth per arch to support the oral appliance;
− Healthy temporomandibular joints and muscles of mastication;
− No history of systemic conditions affecting periodontal health (e.g., uncontrolled diabetes);
− No smoking habit or substance abuse;
− No severe xerostomia;
− No history of recent antibiotics use, pregnancy, or known allergies affecting the treatment.

The full set of inclusion criteria is shown in Supplementary File S2. Subject evaluation, data collection, and clinical procedures were performed in the clinics of the Department of Periodontology and Sleep Research Program, Texas A&M University College of Dentistry, Dallas, Texas. The research coordinator, ZG, obtained consent from and enrolled the patients and prepared the envelopes to assign the interventions as described below.

#### 2.1.2. Clinical and Microbiological Assessments

After performing full-mouth periodontal charting, 10 deeper periodontal pocket sites, five from the left side of the mouth and five from the right side, were selected to collect subgingival plaque samples at baseline and again at 12 weeks using sterilized absorbent paper points, size 30/0.04 taper (Patterson Dental, St. Paul, MN, USA). These samples were analyzed using polymerase chain reaction (PCR) to detect bacterial DNA, measuring quantitative changes in bacterial composition and propagation during the observation period. The analysis was conducted by OralDNA Labs (Eden Prairie, MN, USA).

#### 2.1.3. Randomization and Intervention

**Figure 1:**
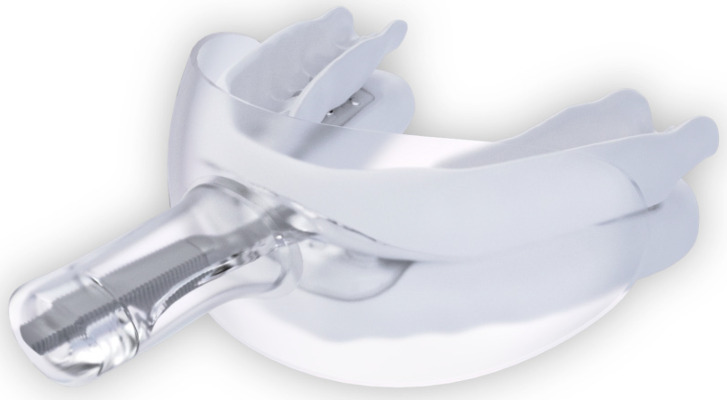
myTAP oral appliance with the mouth shield in place. (sourced from Airway Management, Inc., Farmers Branch, TX, USA).

**Figure 2:**
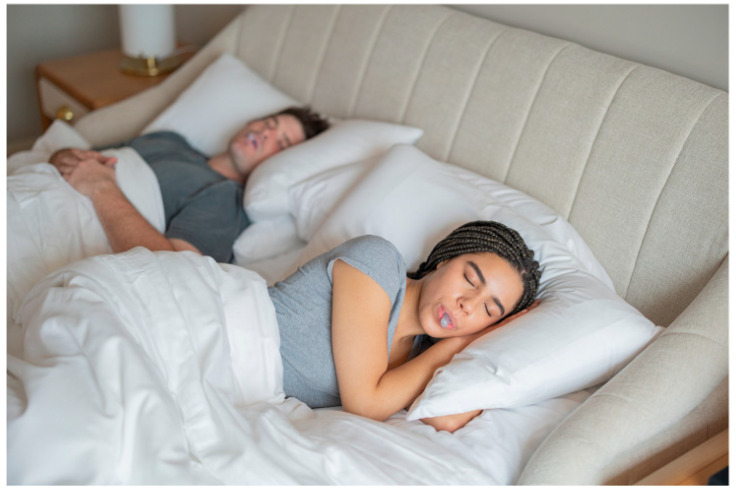
A patient wearing a myTAP oral appliance. (sourced from Airway Management, Inc.).

The randomization sequence regarding which mouth side received SRP initially was produced using Online Research Randomizer software, Version 4.0, by ES. At 2 weeks, all participants were fitted chairside with a self-titrated myTAP^®^ oral appliance (AMI Inc. Carrollton, TX, USA; Figure 1 and Figure 2) by a boarded sleep medicine dentist (JH).

#### 2.1.4. Periodontal Examination

The blinded examiner (C.P.C.), a periodontist, performed all the periodontal assessments, while another periodontist (J.Y.L.) conducted the treatment and data collection. Periodontal measurements were obtained using a UNC 15 mm periodontal probe (Devemed GmbH, Tuttlingen, Germany) and measured all the teeth present on six sites per tooth per subject:

− Probing pocket depth (PPD): Distance from the free gingival margin to the base of the pocket;
− Gingival margin position (GM): Distance from the cementoenamel junction (CEJ) to the free gingival margin;
− Clinical attachment level (CAL): Distance from the CEJ to the base of the pocket;
− Bleeding on probing (BOP): Percentage of total sites with bleeding;
− Plaque Index (PI): Percentage of total sites with plaque;
− Pocket depth greater than 4 mm (PD4): Percentage of sites with probing pocket depths > 4 mm.

Differences between the baseline (T0) and 12-week (T3) measurements were also computed.

After each subject’s initial periodontal assessment and data collection, J.Y.L. was given an envelope containing the randomly assigned mouth side to receive the SRP treatment. Scaling and root planing was performed by J.Y.L. under local anesthesia at the assigned sites on the designated side. Assigned sites on the contralateral side (non-SRP side) were given superficial supragingival polishing (for aesthetic purposes only), avoiding the subgingival sulcus. Standard oral hygiene instruction and verbal reinforcement were also provided. These are known to have a minimal effect on the periodontal outcomes measured here [42].

### 2.2. Ethical Considerations

Written informed consent was obtained from all participants. The study was approved by the Institutional Review Board at Texas A&M University College of Dentistry on 26 January 2023 (IRB2022-1450-CD-FB) and registered at ClinicalTrials.gov (NCT06797089). To ensure all participants received the standard of care, SRP was provided on the untreated side *after* the study period, with retreatment of the treated side, if necessary. The informed consent document is in Supplementary File S3.

### 2.3. Oral Appliance

Subjects were given instructions on how to use their oral appliances during sleep. Oral appliance therapy was started at approximately 60% of the subject’s maximum protrusive position. Oral and written instructions were given to each subject on how to adjust the OA per the manufacturer’s directions. After acclimating to the OA’s initial position, patients were instructed to turn the mandibular advancement mechanism up to 2 turns (0.25 mm each) each night if OSA events, snoring, or daytime sleepiness persisted. They were instructed to delay advancements if they experienced discomfort. The subjects began using the mouth shields at 8 weeks. The clinicians used the 4-week and 8-week sleep-recording results as guides to instruct the subjects to further advance their OAs, if needed.

### 2.4. Home Sleep Test

Home sleep recordings were made at baseline (T0, one night) to confirm the presence of MB, snoring, and OSA events and to assess their severity. Two consecutive-night recordings were attempted at time points T1–T3. Data from each of the two recording sessions, when available, were averaged to determine night-to-night variability. Respiratory dynamics data, including RDI, were obtained and analyzed using Noxturnal software (US version 6.3.2.34938) and the NOX T3 recorder (NOX Medical, Reykjavík, Iceland). A Nonin finger probe pulse oximeter was used to measure oxygen saturation. Each subject was instructed as to how to use the recorder and how to self-apply the sensors. A recording of at least five hours without artifacts from all channels was considered acceptable. Compliance was defined as completing the study protocol from baseline to 12 weeks using the assigned intervention >5 h/night as self-reported at every visit. Apnea and hypopnea events were visually scored using the revised American Academy of Sleep Medicine 2007 scoring criteria [43]. RDI was defined as the sum of all apneas and hypopnea events/hour (apneas, >90% reduction in airflow from the baseline; hypopneas, 30–90% airflow reduction from the baseline associated with ≥3% oxygen desaturation and duration ≥10 s). The hypopnea index (#events/h) used similar hypopnea event criteria. Respiratory rate (#breaths/minute), the oxygen desaturation index (ODI, #events/h with ≥3% oxygen desaturation), SaO_2_ (percent), MB (#minutes; ≥3 breaths minimum duration ≥20 dB), and snoring percent (snore minutes ≥20 dB/analysis duration minutes) were obtained. The RDI includes the number of respiratory effort-related arousal (RERA) and snoring RERA events per hour of recorded time rather than sleep time, so may slightly underestimate the apnea hypopnea index (AHI). RERA scoring used ANS arousals based on pulse signal heart rate increases of ≥5 beats/min in addition to a sequence of breaths lasting ≥10 s, characterized by increased respiratory effort or by flattening of the inspiratory portion of the nasal pressure signal. Sleep measured by electroencephalography was not obtained during the HST, thus justifying the use of the RDI as the more appropriate measure to assess OSA severity. Snoring and MB sounds were manually scored if they were in synchrony with breathing effort and exceeded the level of background noise using the NOX T3 built-in audio sensor [44]. Differentiation between nasal snoring without MB versus snoring with MB relied on snore pattern recognition in the audio and audio volume (dB) signals. Nasal snoring presents with a crescendo–decrescendo “diamond shape” type pattern in the audio signal and a plateau peak in the audio volume (dB) signal. Both of these signal characteristics are absent during MB. The recorder placement followed the manufacturer’s recommended mid-thoracic mounting. The sleep recordings were collected, scored, and entered into the database by PS; he was blinded with regard to which intervention the subjects had received.

### 2.5. Statistical Analysis

R Studio Statistical package (version 1.4.1103) and SPSS v29 software (IBM Inc., Chicago, IL, USA) were used for statistical analysis. The majority of the sleep respiratory variables were not normally distributed, so frequencies, medians, and interquartile ranges (IQR) were used for description. To determine possible differences between the two treatment groups in ancestry, Fisher’s exact test was used. For identifying significant between-group differences for continuous variables, Mann–Whitney U tests were used. To evaluate within-group differences, Wilcoxon’s signed-rank tests were used. The binomial test was used to test whether the proportion of subjects positive for the bacterial species of interest changed from the baseline. A significance level of *p* < 0.05 was used for the analyses.

## 3. Results

### 3.1. Subjects

**Figure 3:**
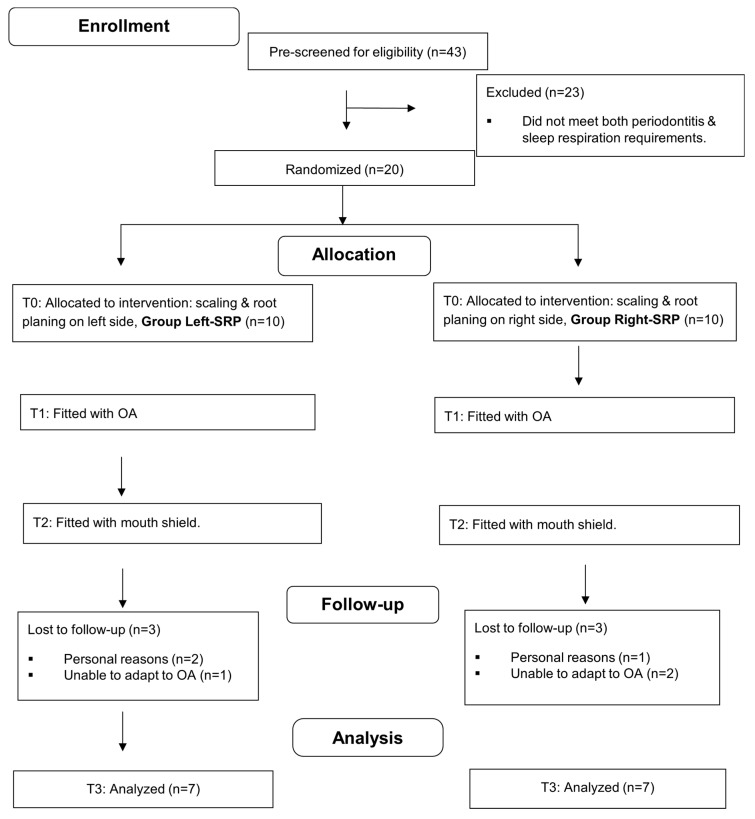
CONSORT flow diagram with attrition; OA, oral appliance.

Forty-three adults were prescreened for enrollment. Of these, 23 were ineligible due to not meeting *both* the periodontitis requirement and the sleep respiration requirements (>4 snores per hour and confirmed MB) from the initial HST. Six of the twenty subjects who received SRP and were fitted with an oral appliance withdrew from the study (two for personal reasons and four due to being unable to adapt to using the OA). Fourteen participants completed the 12-week study; their demographics and periodontal parameters are displayed in Table 1. There were no statistically significant differences between the two treatment groups (initial SRP side) regarding age, BMI, gender, ancestry, or any of the periodontal variables at baseline (Mann–Whitney tests, Fisher’s exact test, and binomial tests used). There were also no differences between sides within subjects at baseline (Wilcoxon tests). The patients’ interaction in the study ran from 3 May 2023 until 2 November 2024. No additional subjects were enrolled past this time due to budgetary and time constraints. The CONSORT subject flow and attrition diagram is shown in Figure 3, and the CONSORT checklist is shown in Supplementary File S4.

### 3.2. Periodontal and Dental Assessments

The median differences from the baseline measurements to T3 are summarized in Table 2, Table 3 and Table 4. No significant differences were observed in any of the periodontal variables except BOP between the SRP and untreated sides of the mouth at T3, both with OAT+ therapy. The percentage of bleeding sites decreased significantly at more SRP sites than in the OAT+ only group (*p* = 0.028). Decreased median percentage values of PD4 and BOP from the baseline were observed for both SRP-treated and non-SRP-treated sites but did not achieve significance (*p* = 0.117–0.196). Microbial data at T3 demonstrated increased median total bacterial counts; however, the *relative contributions* of the red, orange, and green complex remained similar to the baseline. *T. denticola* increased significantly in the SRP group (*p* = 0.043) at T3. The presence versus absence of the three species at baseline versus T3 is shown in Table 5. The absence of *P. gingivalis* among the total sites at T3 was significant (*p* = 0.0135), as was that for the non-SRP sites for *T. forsythia* (*p* = 0.0188).

### 3.3. Sleep Respiration Assessment (Table 6)

At T3, none of the respiratory variables differed significantly between the two groups as defined by which side initially received SRP. The following results are for both groups combined.

**Table 6:** Sleep respiration: Comparison between baseline and T3, all subjects (n = 14).

Variable	Baseline	T3	*p*-Value
Mouth Breathing (%)	8.9 [2.1–36.1]	1.9 [0.3–8.1]	0.022
Respiratory Disturbance Index (# events/h)	22.1 [15.0–28.1]	12.6 [5.1–17.6]	0.009
Respiratory Disturbance Index—Supine (# events/h)	27.9 [21.7–39.0]	12.7 [4.5–19.1	0.002
Oxygen Saturation (%)	92.6 [91.2–92.9]	92.2 [91.6–93.2]	0.782
Oxygen Desaturation Index (# events/h)	19.2 [18.0–22.5]	13.2 [5.9–16.5]	0.025
Oxygen Desaturation Drop (%)	5.1 [4.2–7.4]	3.8 [3.5–4.3]	0.025
Snoring Percent (%)	26.1 [8.1–53.7]	4.5 [1.9–10.3]	0.010
Respiratory Rate (breaths/m)	13.8 [12.8–15.5]	14.8 [13.6–15.9]	0.280

Abbreviations: IQR, interquartile range. Median [25th–75th percentile].

### 3.4. Mouth Breathing

MB was significantly decreased (*p* = 0.022) with OAT+ at T3 compared with the baseline.

### 3.5. Respiratory Disturbance Index (RDI) and RDI–Supine

The RDI (*p* = 0.009) and the RDI–supine (*p* = 0.002) were significantly lower with OAT+ at T3 compared with the baseline.

### 3.6. SaO_2_ and Oxygen Desaturation Index (ODI)

When compared with the baseline, SaO_2_ was not significantly changed at T3 with OAT+ (*p* = 0.782). The ODI (*p* = 0.025) and oxygen desaturation drop (%) were decreased with OAT+ at T3 compared with the baseline (*p* = 0.025).

### 3.7. Snoring Percent (%)

The snoring % was significantly lower (*p* = 0.010) with OAT+ at T3 compared with the baseline.

### 3.8. Respiratory Rate (Breaths/Min)

The respiratory rate was not significantly different at T3 with OAT+ (*p* = 0.280) compared with the baseline.

## 4. Discussion

This study sought to compare periodontal and sleep respiratory outcomes in patients with periodontitis and OSA after being treated with OAT+ therapy, with and without SRP. A split-mouth design was used to isolate the effects of SRP under OAT+ and minimizing inter-subject variability by having each participant serve as their own control. The scope of the study was to observe any potential changes in periodontal health among mouth breathing OSA subjects using OAT+ rather than as a potential periodontal treatment. The research population focused on adults with mild to moderate generalized periodontitis (Stage I–II, Grade A–B) who presented at baseline with MB, snoring, and OSA. Given the study’s relevance to both periodontology and sleep medicine, simplified and current classification terminology was used to enhance accessibility and understanding across both disciplines. The results provide interesting insights and an interdisciplinary approach to consider OAT+ as adjunctive therapy for periodontitis, albeit with certain limitations.

OSA during sleep may lead to systemic physiological dysfunction and dysbiosis of the oral microflora and more periodontal inflammation [19], particularly when exacerbated by MB. Preventing MB and encouraging nasal breathing promotes ventilation efficiency while reducing snoring [45,46], respiratory resistance [47], apneas and hypopneas [48], airway collapsibility [49], and blood oxygen saturation decreases [50]. Since MB is known to reduce the success of SRP, its prevention should also enhance treatment outcomes [51].

The absence of significant differences in periodontal variables between the SRP-treated and untreated sides at 12 weeks suggests that OAT+ provided comparable improvements to the SRP treatment within this timeframe. When compared with the baseline, both groups showed a slight improvement in most of the periodontal parameters: a decreased percentage of deep pockets and percentage of BOP sites. These occurred despite slight increases in plaque accumulation. Importantly, we found that the majority of the *P. gingivalis*-positive sites became negative (under-detected limit) at the end of the study for both sides. This finding aligns with previous studies that demonstrated that *P. gingivalis* responded well to non-surgical intervention, while the *T. forsythia* percentage remained substantial (slightly increased). The reduction in *P. gingivalis* after scaling and root planing has been reported to be about 70%. In contrast, the reduction in *T. forsythia* was far less: 24% after non-surgical treatment [52]. The presence of bacteria alone may not be the determining factor in disease progression, as these species have been found in both diseased and healthy sites [53]. However, the absence of or reduction in keystone pathogens is an encouraging outcome, especially for susceptible individuals, as it may help reduce the progression of their periodontitis. It is known that species such as *T. denticola,* which have the ability to infect gingival epithelial cells [54], can readily recolonize within the first 4–8 weeks after SRP [55,56]; this red complex species was found to have increased significantly at the 12-week observation. Because of its invasive mode of action (in contrast to *P. gingivalis*), it may be less susceptible to attenuation by the oral environment improvement provided by the mouth shield (at 8 weeks into the study). Nevertheless, there was more reduction in the number of bleeding sites at T3 in the SRP group than in the non-SRP group. The reduction in bleeding on probing indicated reduced inflammation and is a good indicator of periodontal stability. It is well established that keeping the percentage of BOP sites at 20% or less lowers the risk for future probing attachment loss [57]. A recent study indicated that sites treated with SRP, despite demonstrating clinical improvement, still had a relatively greater abundance of pathogenic species and fewer nitrate-reducing genera, when compared with the healthy sites. The dysbiosis of the oral microbiome may still persist, even after disease remission [58]. Our results emphasize the importance of SRP as a fundamental periodontal therapy via the mechanical removal of the irritants and pathogens. The inflammatory response still varied individually, and further evaluation of the accompanying local and systemic biomarkers may enhance future studies.

Contrary to our expectations, we were unable to show an association between reductions in the RDI and improvement in the periodontal parameters among the OAT+ responders (those with a RDI reduction of more than 50%). This could be due to the predominantly mild periodontal condition of this sample (mostly Stage I-II, mild periodontitis); the outcomes of the SRP resulted in insignificant changes compared with what is expected for moderate to severe patients with deeper pocket depths of more than 6 mm. Tooth-related factors, such as restoration type (bridge, crown, implant restoration) and root form (muti-rooted vs. single-rooted) may have confounded the outcomes [59,60]. We found no significant difference between groups at baseline in terms of pocket depth, bleeding sites, or plaque distribution, so the differences that were found at the end of the study can be attributed to treatment effects. While cross-contamination between non-SRP and treated sites cannot be entirely ruled out, our findings align with previous studies, indicating that oral appliance therapy does not negatively impact periodontal health within this 12-week experimental period [61].

The absence of a negative control group limited the ability to isolate the effects of OAT+ on periodontal outcomes, potentially impacting interpretation of the OAT+ results. An alternate and potentially stronger design would have been a parallel group design, both with full-mouth SRP but only one group with OAT+; for ethical reasons, we chose not to deny any subjects treatment for their OSA, a serious and life-threatening disease. Nonetheless, the observed reduction in MB with OAT+ suggests that the therapy helped mitigate certain OSA-related behaviors that contribute to periodontal issues, despite 43% of the subjects still exhibiting MB at 12 weeks. This subgroup may have experienced residual MB due to suboptimal appliance titration; their 12-week RDI was nearly double that of non-MB subjects (12 vs. 7 events/h). Alternatively, this finding may reflect a mouth breathing endotype. In evaluating mouth breathing in OSA patients using drug-induced sleep endoscopy, Yang et al. [62] found that mouth closure increases overall airflow in the *majority* of patients. This is consistent with our findings regarding mouth shield use. However, they also found that a subgroup with high levels of mouth breathing during sleep and velopharyngeal obstruction (22%) had *reduced* airflow with mouth closure; the other subgroups had adequate airflow with mouth closure. Future studies evaluating OAT+ effectiveness should consider such endotype heterogeneity regarding the oral–nasal breathing spectrum. Additionally, some patients were on medications for hypertension, hypercholesterolemia, etc., which may cause hyposalivation and reduce the efficacy of OAT+ in managing MB [63]. Finally, the treatment success of mandibular advancement device therapy depends highly on compliance and is often related to patient-centered outcomes such as the perception of symptom improvement [64]. Although we attempted to survey the subjects on comfort-related outcomes, the response rate precluded formal analysis n = 8). Tooth, gum, and jaw pain; dry mouth; and drooling trended downwards among the respondents; perceptions of changes in the bite did not change. Larger-scale studies should evaluate such perceptions.

The significant reductions in overall and supine RDI with OAT+ indicate effective mitigation of OSA severity, especially in the supine position, consistent with previous findings [63]. These improvements suggest enhanced upper airway patency during sleep, likely reducing systemic inflammation and OSA-related hypoxia. The absence of significant SaO_2_ changes also aligns with earlier work [63], which found that OAT+ caused less SaO_2_ variation than OAT alone. Notable reductions in ODI and oxygen desaturation further reflect improved nocturnal oxygen stability. Additionally, decreased snoring time highlights OAT+’s efficacy in enhancing airway patency and airflow, and reducing turbulence.

Though the research yielded promising data, it was not without limitations. The 12-week study may be too short for some OSA patients with MB to fully self-titrate OAT+ for optimal airway patency. Our small cohort, characterized by mild to moderate periodontitis, OSA, and MB, limits the statistical power and generalizability, highlighting the need for larger studies. While 14 subjects were sufficient to show significant respiratory improvements, the study was underpowered to detect changes in periodontal variables such as PD4. Post hoc power analysis showed effect sizes (dz) of around 0.40 for near-significant findings, suggesting at least 43 subjects are needed to detect small effects statistically. Forthcoming research should test improved mouth shield designs to better reduce MB and assess the direct effects on periodontal health. Combining OAT+ with SRP may aid in managing periodontitis in OSA patients with MB, but confounding factors such as natural variation, compliance, restorations, and plaque control require larger samples to account for variability. The results and their interpretation must be viewed with caution due to the limitations of the design; the small, older sample; and potential cross-contamination in the split-mouth design. A design with OAT+ as the independent variable may lead to more conclusive results. Future studies should also consider direct inflammatory markers like salivary cytokines in addition to the oral microbiome; while it is related to periodontitis, it alone may not be the best indicator of periodontal health or disease progression. Our findings apply to older adults with mild–moderate periodontitis; OAT+ should be used cautiously in severe cases due to periodontal instability and limited impact on *T. denticola*. Such patients should follow more frequent periodontal maintenance.

## 5. Conclusions

This is the first study to evaluate the joint effects of OAT+ and SRP in OSA patients with MB and periodontitis. After 12 weeks, there were no major differences between OAT+ alone and OAT+ with SRP in most periodontal clinical parameters, suggesting OAT+ may serve as a nocturnal adjunctive therapy for mild to moderate periodontitis in this population. While OAT+ in conjunction with SRP significantly reduced *P. gingivalis*, changes in other microbial flora were less clear. OAT+ also reduced overall and supine RDI and snoring time, and may support chemo-reflex stability, as indicated by reductions in ODI and oxygen desaturation drop (%).

## Figures and Tables

**Table 1:** Subject characteristics by group assignment at baseline.

Characteristics	Right-SRP (n = 7)	Left-SRP (n = 7)	*p*-Value
Age (years)	72.0 [60.5–75.0]	63.0 [55.0–67.5]	0.178
BMI (kg/m^2^)	26.7 [25.5–32]	27.6 [27.1–28.4]	0.847
Gender (# [%] Male)	5 [71.4]	4 [57.1]	1.000
Ancestry (# [%])	European: 5 [71.4]	European: 3 [42.9]	0.592
	African: 2	African: 2; Hispanic: 1 Asian: 1	
PD4 (%)	3.08 [1.83–5.05]	3.97 [2.3–4.91]	0.534
BOP (%)	4.495 [1.8–8.13]	5.66 [2.75–10.12]	0.221
PI (%)	30.53 [23.58–39.16]	27.7 [25.69–35.52]	0.778
PPD (mm)	2.4 (2.21–2.66)	2.38 (2.21–2.58)	0.975
CAL (mm)	2.19 (1.89–2.6)	2.27 (1.89–2.53)	0.753

Treatment groups are designated according to which side initially received SRP (scaling and root planing), by random assignment. Values are medians [interquartile range, i.e., 25th–75th percentile]; BMI, body mass index; PD4, % of sites with probing depths ≥ 4 mm; BOP, % sites with bleeding on probing; PI (%), plaque percentage; PPD, probing pocket depth; CAL, clinical attachment level.

**Table 2:** Periodontal variables by treatment side before and after treatment; all subjects received OAT+.

Variables	SRP (n= 14)	NSRP (n = 14)	*p*-Value (SRP vs. NSRP)
PD4 [%]			
*Baseline*	3.08 [1.83–5.057]	3.97 [2.30–4.907]	0.534
*T3*	2.275 [1.025–2.62]	2.275 [1.04–4.57]	0.328
*p*-value [Baseline vs. T3]	0.170	0.117	
BOP [%]			
Baseline	4.495 [1.88–8.13]	5.66 [2.75–10.11]	0.221
T3	2.82 [1.70–5.035]	4.01 [2.88–6.16]	**0.028**
*p*-value [Baseline vs. T3]	0.117	0.196	
PI [%]			
Baseline	30.53 [23.58–39.16]	27.77 [25.69–35.52]	0.778
T3	29.69 [25.03–31.54]	29.76 [27.01–33.20]	0.177
*p*-value [Baseline vs. T3]	0.402	0.925	

SRP, scaling and root planing; NSRP, no scaling and root planing; OAT+, oral appliance therapy plus mouth shield. T3, 12 weeks. Values are medians [interquartile range, i.e., 25th-75th percentile]). PD4, % of sites with probing depths ≥ 4 mm; BOP, % of sites with bleeding on probing; PI, plaque percentage.

**Table 3:** Oral flora complexes, percentages by treatment side. All subjects received OAT+.

Oral Flora Complex				*p*-Value SRP vs. NSRP
		SRP	NSRP	
Red Complex (%)				
	Baseline	2.86 [1.27–9.81]	8.47 [1.43–18.82]	0.454
	T3	3.18 [0.47–15.32]	8.94 [0.39–29.06]	0.482
	*p*-value (Baseline vs. T3)	0.875	0.551	
Orange Complex (%)				
	Baseline	57.89 [46.67–66.75]	61.30 [33.54–66.59]	0.701
	T3	49.69 [43.19–60.57]	58.68 [33.72–67.42]	0.804
	*p*-value	0.363	0.925	
Green Complex (%)				
	Baseline	37.26 [18.61–43.28]	32.66 [25.45–42.62]	0.982
	T3	42.51 [19.92–49.64]	29.51 [14.96–39.72]	0.454
	*p*-value	0.778	0.683	
Total Bacteria Count				
	Baseline	16,135,698 (3,008,356–23,329,026)	18,316,978 (8,375,603–23,367,001)	0.177
	T3	19,486,858 (8,431,788–25,114,553)	288,609,082 (11,846,659–34,644,254)	0.363
	*p*-value	0.551	0.363	

SRP, scaling and root planing; NSRP, no scaling and root planing. The bacterial complexes are shown as a percentage of the total bacterial counts as samples from that side of the mouth. Values are medians [interquartile range; i.e., 25th–75th percentile].

**Table 4:** Oral flora species, counts by treatment side. All subjects received OAT+.

Red Complex Count				*p*-Value
		SRP	NSRP	
*P. gingivalis*	Baseline	0 (0–147,945)	0 (0–392,564)	0.685
	T3	0 (0–0)	0 (0–315,706)	0.582
	*p*-value	0.463	0.866	
*T. forsythia*	Baseline	122,752 (14,906–1,501,042)	187,298 (27,884–2,637,067)	0.734
	T3	100,937 (1974–1,779,789)	324,444 (0–2,602,153)	0.908
	*p*-value	0.861	0.552	
*T. denticola*	Baseline	0 (0–32,543)	0 (0–61,902)	0.734
	T3	0 (0–317,398)	0 (0–473,665)	0.839
	*p*-value	**0.043**	0.173	

Values are medians [interquartile range; i.e., 25th–75th percentile].

**Table 5:** Presence of oral flora species by sides.

*P. gingivalis*			
	SRP	NSRP	Total
Baseline	5/14	6/14	11/28
Final	2/14	3/14	5/28
*p*-Value	0.076	0.085	0.0135
*T. forsythia*			
	SRP	NSRP	Total
Baseline	12/14	13/14	25/28
Final	11/14	10/14	21/28
*p*-Value	0.3223	**0.0188**	0.039
*T. denticola*			
	SRP	NSRP	Total
Baseline	5/14	6/14	11/28
Final	6/14	6/14	12/28
*p*-Value	0.801	0.609	0.920

## Data Availability

The raw data supporting the conclusions of this article will be made available by the authors on request.

## Supplementary Materials

The following supporting information can be downloaded at: https://www.mdpi.com/article/10.3390/dj13070292/s1, File S1: Timetable of subject activities; File S2: Inclusion criteria; File S3: Informed consent document; File S4: Consort checklist.
