# Supplementary material for: Effects of Oral Appliance Therapy with a Mouth Shield in Periodontitis Patients Who Snore: A Split-Mouth Randomized Controlled Trial
Source: Dent J (Basel). 2025 Jun 27;13(7):292. doi: 10.3390/dj13070292 (PMC12294119; doi:10.3390/dj13070292)
Supplement: Supplementary file 1 [file dentistry-13-00292-s001.zip › dentistry-3580690-supplementary/dentistry-3580690-supplementary/File 1. Activity table 1 for ICD final 4-23-25.pdf]

Timetable of patient activities including interventions, clinical, sleep and microbial evaluation time-points.

Time Point:	Pre-screening	Screening & Baseline: T0	T1	T2	T3	Post-experiment
**Visit Number:**	**1**	**2**	**3**	**4**	**5**	**6**
Week Number:	1	2	4	8	12	12-13
Length of visit:	1 hour	3 hours	1 hour	45 min.	1 hour	2.5 hours
Activity						
Informed Consent	X					
Medication & Med. History	X					
Sleep Recording	X		XX	XX	XX	
Radiographs (X-rays) taken	X					
Initial Dental Exam for OA		X				
OA Fitting & Instruction			X			
Begin using Mouth Shield				X		
Oral Hygiene Instruction		X				
Full Mouth Periodontal & Dental Exam		X			X	
Periodontal Periodic exam			X	X		
Scaling & Root Planing		Half mouth				Full mouth
Subgingival Microbe Collection		X			X	
Comfort Questionnaire		X	X	X	X	

OA, Oral appliance. XX, 2 nights of sleep recordings. Last column is complimentary care provided after completion of study
