# Supplementary material for: Effects of Oral Appliance Therapy with a Mouth Shield in Periodontitis Patients Who Snore: A Split-Mouth Randomized Controlled Trial
Source: Dent J (Basel). 2025 Jun 27;13(7):292. doi: 10.3390/dj13070292 (PMC12294119; doi:10.3390/dj13070292)
Supplement: Supplementary file 1 [file dentistry-13-00292-s001.zip › dentistry-3580690-supplementary/dentistry-3580690-supplementary/File 2. myTAPplus Perio ICD APPRV 2-17-23.pdf]

Texas A&M University School of Dentistry
Dallas, Texas

Informed Consent to Participate in a Research Study

Study Title: *myTAP* Oral Appliance Plus Mouth Shield to Reduce Periodontitis in Mouth-Breathers who Snore.

Short Title: Periodontitis Attenuation with OA+ Therapy in Mouth-breathers who Snore

Principal Investigators: Emet Schneiderman, PhD; emet@tamu.edu, 214-828-8377
Co-Principal Investigator: Preetam Schramm, PhD; schramm@tamu.edu
Co-investigators: Ju Ying Lin, DDS, MS, ; jlin01@tamu.edu ; Jason Hui, DDS; William Stenberg DDS, PhD

Research Coordinator & Emergency Contact: Zohre German, MS; german@tamu.edu, 214-828-8291.

Funded by: Texas A&M University Health Science Center, VP Seedling Program

To Participants:

This research study is investigator-initiated. Please read the following document as a part of the informed consent process. The informed consent process is to advise you of the risks and benefits involved in the study so you can freely decide whether to participate or not. Informed consent includes having the study fully explained to you, an opportunity to ask questions, have your questions answered to your satisfaction and obtain a written copy of the informed consent document to read, retain and sign.

Description of Study:

You are being invited to participate in this research study because you may have periodontal disease, mouth breathe and snore. It will take place in clinics of the Texas A&M University School of Dentistry, specifically in Sleep Research Program Clinic, Rooms 20 and 725 at 3302 Gaston Avenue and the Clinical building at 3000 Gaston Avenue, Dallas TX 75226.

a. Study purpose

The purpose of this pilot study is to determine whether snoring, mouth breathing, and periodontitis can be reduced by wearing the myTAP oral appliance (OA) with a mouth shield (OA+) during sleep. This is an interventional study to objectively evaluate OA+ therapy on breathing during sleep, sleep quality, and whether it can improve problems related to mouth breathing such as dry mouth, bleeding and inflammation of the gums and tissues that support the teeth (gingivitis and periodontitis). If you are 18 years of age or older, you snore and mouth breathe, have at least 8 teeth in your upper dental arch, and your oral health is adequate to wear an OA, you may be eligible to enroll. You will be expected to wear the OA+ (Figure 1) for at least 12 weeks.

b. Periodontal Evaluation & Treatment

An experienced periodontist (dental specialist on gums and tissues that support the teeth) will examine your teeth before starting OA, and after 4-, 8- and 12-weeks. The periodontist (PD) will record several measures of gum health, including bleeding upon probing with a dental instrument. Depending upon the extent of your periodontitis, the PD will provide scaling of the crowns of the teeth and possibly deep cleaning of the roots (root planing) to establish good periodontal health, the <u>standard of care</u> for periodontal disease. This study uses what is called a split mouth design, where one side of your mouth will receive periodontal treatment at the beginning of the study, and the other, after its completion at 12 weeks. You will be randomly assigned to begin with either the

right or left side. The randomization will be done using a procedure like flipping a coin. The PD will conduct follow-up assessments at 4-, 8-weeks and 12-weeks. Also at 12 weeks, saliva, plaque, and left-over debris from the cleaning be collected. The saliva is being collected to measure the amount of cortisol (a stress hormone) in it. The material on the probing instrument is being collected to measure bacterial deoxy nucleotide acid (DNA). From these specimens we will be able to look for changes in inflammation and identify the specific bacteria in your mouth that relate to periodontal disease. The initial and final appointments (at 12-13 weeks) for scaling and root planing will take up to 3 hours each. The other 5-6 appointments will be 45 to 60 minutes long. The procedures above are part of the periodontal standard of care at the Texas A&M University School of Dentistry with the following exceptions, which are research-only procedures: (1) the split-mouth design involving the delaying of treatment for one half of your mouth to 12 weeks, (2) additional periodontal exams [about 10 each] at 4 and 8 weeks, and (3) saliva and plaque collection. One or 2 additional appointments are needed beyond the standard 5-6 appointments for the research-only activities.

c. Home sleep recordings

Over the same 12-week period described above, you will do home sleep recordings at regular intervals at 4 to 5 time points (see Table 1). At each of these time points you will do two consecutive nights of sleep recording. The sleep recording device includes a small cuff worn on a finger and a wristwatch-like device to measure your blood oxygen level during sleep. To evaluate your breathing, you will be asked to wear a nasal pressure cannula, a comfortable belt around your chest and another around your abdomen. You will be asked to attach two adhesive electrodes to your chest to collect your heartbeat data during sleep. The heartbeat data will be analyzed to determine your sleep quality. Snoring data and the presence or absence of mouth breathing will be collected from a microphone on the recording device. We believe that improvements in sleep may relate to improvements in the periodontal measures mentioned above.

c. Questionnaires

In addition to the sleep recordings at several time points, subjects will complete several brief comfort questionnaires. They will ask about sleep, oral comfort/discomfort etc.

d. Oral appliance plus mouth shield (OA+)

The OA will be fitted by one of the expert team dentists in a single appointment. The adjustable OA consists of two plastic plates that cover the upper and lower teeth. The OA acts by moving your lower jaw forward to increase the space in the back of your mouth (upper airway/throat). The increased space is expected to improve breathing during sleep and lessen snoring. This well-established OA design includes a midline screw apparatus that allows the user, under close dentist supervision, to gradually bring the lower jaw forward to a position that optimizes your airway. At the four-week appointment you will receive the mouth-shield (MS), a thin silicon device that fits over the OA. The OA plus the MS is called OA+. The saliva that is produced in your mouth protects your teeth, gums, and other tissues in your mouth; the MS will help to keep saliva in your mouth and may minimize dry mouth.

In addition, all participants will be given the opportunity to use and keep their OA+ after the 12-week experimental period. To help you adjust the OA to an optimal position, you will receive information from the Study Coordinator about the results of your sleep recordings and if necessary, suggestions to advance the OA to a new position to improve your breathing during sleep. The study dentists will provide optimal dental care and evaluate your oral health before starting OA treatment and at the at 4- and 8-weeks and at 12-week visits.

IRB NUMBER: IRB2022-1450-CD-FB
IRB APPROVAL DATE: 03/02/2023

Justification for the trial: Why is this study being done?

Breathing (respiration) is one of the body's vital functions and under normal conditions is done through the nose. When breathing is mainly done through the mouth instead of the nose, it is called mouth breathing (MB). Snoring and interruptions in breathing during sleep (obstructive sleep apnea) are frequently observed in MB, regardless of age. MB impairs oral health, reduces quantity and quality of saliva, and increases dry mouth, risk of developing dental caries, gingival inflammation, periodontitis, bad breath, and dry lips.

Therefore, the purpose of this study aims to evaluate whether the OA+ can improve snoring, breathing (respiration) during sleep and reduce periodontitis. Mouth breathing interferes with sleep, so it is expected that the mouth shield may lessen this interference. Using the mouth shield is expected to lead to healthier gums and teeth, as well as reduced oral discomfort from dry mouth.

The purpose of collecting sleep data with OA use is to determine its success in promoting stable respiration during sleep and reducing the number of events in which breathing stops (apneas) or diminished (hypopneas) and if the amount of oxygen in the blood is low (oxygen desaturation).

The sleep recorder (NOX T3, Nox Medical, Reykjavík, Iceland) is a Food and Drug Administration (FDA) cleared and CE marked. The oral appliance including the mouth shield (myTAP, AMI Inc., Dallas Texas) is currently marketed as a medical device to treat snoring and obstructive sleep apnea and is FDA cleared.

Why am I being asked to participate in this study?

You are being asked to participate in this study because you stated you snore and mouth breathe, and a team dentist has confirmed your periodontitis. We will give you a home sleep test to confirm if snoring and MB is present. You may be eligible to enroll in the study, based on the other inclusion criteria. If you have stable cardiopulmonary disease (heart failure, Chronic Obstructive Pulmonary Disease, ventricular dysrhythmia), morbid obesity or other serious health conditions, you must be under a physician's care and obtain written permission from him/her to participate in this study.

How long will the study take?

Approximately 12 weeks.

How many people are participating in this study?

Up to 27 participants are expected to enroll and complete the study.

Figure 1. *myTAP* Oral Appliance plus mouth shield

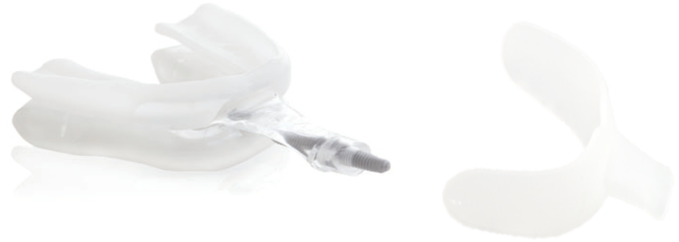

Procedures you will be asked to follow:

Study participants will be required to visit the Sleep Research Program Office at Texas A&M School of Dentistry (SOD) approximately seven or eight times.

IRB NUMBER: IRB2022-1450-CD-FB
IRB APPROVAL DATE: 03/02/2023

Table 1. The number and purpose of visits to the research study center at Texas A&M School of Dentistry.

Time Point:	Pre-screening	Screening & Baseline: T0		T1	T2	T3	Post-experiment
**Visit Number:**	**1**	**2**	**3**	**4**	**5**	**6**	**7**
Week Number:	1	1	1	4	8	12	12-13
Length of visit:	1 hour	3 hours	45 min.	1 hour	45 min.	1 hour	2.5 hours
Activity							
Informed Consent	X						
Medication & Med. History	X						
Sleep Recording	X		XX	XX	XX	XX	
*Radiographs (X-rays) taken	X						
Initial Dental Exam for OA			X				
OA Fitting & Instruction			X				
Begin using Mouth Shield				X			
Oral Hygiene Instruction		X					
Periodontal & Dental Exam		X		X	X	X	
Saliva Collection - Cortisol		AM				AM	
Periodontal Deep Cleaning		Half mouth					Full mouth
Plaque Collection		X				X	
Comfort Questionnaire		X		X	X	X	

- If possible, Visits 1 & 2 will be done in a single day, with informed consent, radiographs etc. in AM and periodontal-related procedures in PM.
- AM: Procedure done in morning, ideally before 10 AM
- OA: Oral appliance
- XX: 2 nights of sleep recordings; participant returns sleep recorder by FedEx
- Blue: Complimentary care provided after completion of study

- *X-rays are taken in the 5th Floor Radiology Clinic, Clinic Building, **3000 Gaston Ave**, Dallas TX 75246; Use front entrance on Gaston Ave.
- All other activities occur in Sleep Research Program office (room 20) and Clinic (room 725) in Texas A&M School of Dentistry, Main Building, **3302 Gaston Ave.**, Dallas TX 75246. Use side entrance on Nussbaumer St.

Table 2. Visit 1 - your requirements

**Item no.**	**Visits 1 & 2 - Processing and Your Requirements**
1	Informed consent
2	Medical / Dental history
3	Medication(s) currently taken
4	Complete questionnaires

During Visit 1, the Clinical Research Coordinator (CRC) will explain the study and answer any questions you may have. The CRC will then ask you additional questions concerning study eligibility. If you are potentially eligible and wish to participate, you will then complete the informed consent process by signing this form. The CRC will

provide you with the Home Sleep Recorder and instruction to record 1 night of sleep. Upon returning the recorder the following day the research team will objectively determine whether or not you snore and mouth breath to further determine your eligibility. You will also have radiographs (X-rays) made of your teeth as the first part of a standard periodontal exam.

During Visit 2, you will be given a periodontal and oral exam by one of the team dentists to assess your teeth, gums, jaw joints and jaw muscles. Your overall health will also be evaluated with a checklist to ensure that you meet all the study inclusion/exclusion criteria. If determined to be adequate, you will then be fully enrolled in the study and your tasks will be explained to you in detail.

At Visit 2, you will provide saliva sample (spit into a tube). Oral hygiene instruction will be provided. You will then undergo a deep cleaning of your teeth on one side of your mouth. This can involve scaling the teeth above and below the gum line, and the roots (root planing). Plaque samples and other waste materials will be scraped from your teeth and saved. This appointment may take up to 3 hours.

On Visit 3 a team dentist will then fit your teeth with the OA which you will take home that day. You will receive instructions how to use and care for the OA. On visit 4 you will receive the Mouth Shield and instructions on its use, to be worn with the OS for the remainder of your mouth.

You will wear the sleep recorder (sensors on your chest; Figure 2) the first two nights (T_0) and record <u>while using</u> your OA. At each of the time points, you will record your sleep for a minimum of five (5) to eight (8) hours each night on 2 consecutive nights <u>with</u> your OA. You will add new adhesive electrodes to record your heartbeat for each recording and place the recorder on your chest at bedtime as shown in Figure 2. The sleep recorder and sensors are removed from your chest/body each morning upon awakening. Additional nights of sleep recordings may be required to optimally adjust the OA, if any of the designated nights of recording fail; a “failed sleep recording” is defined as a night recording less than 5 hours in duration or contains sufficient artifact (“noise”) to make the data uninterpretable. You will also fill out several short questionnaires concerned with sleepiness, snoring and quality of life at time points T_0 to T_3. To help you adjust the OA to an optimal position, you will receive CRC coaching based on suggestions from the sleep specialist to achieve the ideal position of your lower jaw.

In addition to the basic oral exam described above, you will be given a complete periodontal exam (gums and their attachments to the teeth) at T_0 to T_3; each of these exams takes about 30 minutes. Following the standard of care, the periodontist will probe and take measurements of the depths of the pockets between the gums and teeth.

To use the NOX Recorder:

1. Push the start button on the recorder to start recording.
2. Place the oximeter on a finger of the non-dominant hand (that is, if you are right-handed place it on your left hand).
3. Collect a minimum of 5 hours of sleep data.
4. Stop recording by removing the device from your body upon awakening the following morning and discard the used adhesive electrodes. Replace the NOX recorder in its carrying case and return it to the study coordinator.

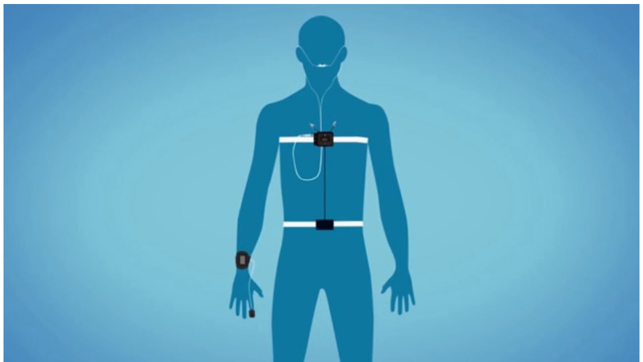

Figure 2. At bedtime, position and attach the NOX recorder over your pajama or T-shirt. If needed, you will use tape to help secure some of the sensors connected to the dev[illegible]

IRB NUMBER: IRB2022-1450-CD-FB
IRB APPROVAL DATE: 03/02/2023

Risks

The minor physical risk associated with the sleep-monitoring aspects of this study are no more than those involved with standard tests of bodily (physiological) functions, for example wearing a heart monitor. The oral appliance is FDA cleared and is in wide use by dental patients throughout the US, Europe and Australia. The OA may cause pain in the jaw joint and teeth, and difficulty in opening or closing the jaw; these conditions are usually temporary. In the long term, the OA may cause changes in tooth position and in the bite (occlusion), as well as damage to the teeth and gums. A morning-aligner device will be custom made for you that helps to minimize changes in the teeth, gums, or occlusion.

The Mouth Shield (MS) has been in use for many years in the marketplace as an accessory to the OA to prevent dry mouth and improve patient comfort. There have been no adverse incidents reported to the manufacturer (AMI) or the FDA. If for any reason you are unable to breathe adequately through your nose, for instance due to a bout of severe nasal congestion, you are to remove the MS. We anticipate that in many individuals, the MS will lessen the nasal congestion, and improve the ability to breathe through the nose. The aspects of this experience that make it “research” rather than “routine clinical care" is that additional physiological and behavioral measurements will be made go beyond what are typically gathered during standard clinical practice.

We do not anticipate any significant physical risk from participating in this study. Though not impossible, we do not expect the adhesive hypoallergenic electrodes used with the NOX recorder to cause itching or rashes. By wearing an OA, a subject may experience some discomfort and/or reduced function of the jaw joint (temporomandibular joint) and muscles of the jaw (chewing muscles) and of the face during and after treatment. Subjects may also experience some reduction in sleep quality due to discomfort while getting used to wearing the OA (adaptation). Other minor risks include temporary irritation of the mouth and oral cavity due to contact with the OA, and excessive salivation or dry mouth from mouth breathing. Repositioning of the lower jaw and tooth movement may also occur but are typically minor and reversible. Also highly unlikely, a subject could swallow or aspirate part of an OA, should it break. Participants will be carefully monitored for all these risks by one or more of the study dentists.

Benefits

Specifically potential benefits from OA+ include

1. improved sleep quality such that it is more stable and restorative (less disrupted/disturbed),
2. an effective treatment for snoring and obstructive sleep apnea that does not require wearing a face mask in which pressurized air is blown through your nose (continuous positive airway pressure or CPAP device),
3. use of a device for reducing snoring that does not require electricity for operation (CPAP), and
4. shifting from mouth to nose breathing to reduce mouth dryness and further improve your periodontal health, beyond the scaling and root planing,.
5. improved quality of life,
6. general health, and well-being, and
7. detailed information on your sleep respiration. For all participants, the researchers or study coordinator will discuss with you possible options that might be of benefit to you if a sleep issue is discovered.

Alternative Treatments:

No alternative treatment options are directly part of this study. If you decide that oral appliance therapy is not right for you, we can refer you to a physician for alternative treatments that may include the use of CPAP. If you have mild to moderate periodontitis and do not wish to participate, you may opt be a regular (non-research patient) in the School's periodontal clinic and receive periodontal standard care (see above) at the standard rates.

Voluntary Participation or Withdrawal: Is my participation voluntary?

Instead of being in this study, you have the choice to not participate. Participation in this study is voluntary. You may quit the study at any time without giving reasons.

Can I participate in another study during this Study?

No, by signing this Consent Form, you confirm that you will not participate in another study during the term of this study that includes the final visit.

Confidentiality: What happens with my data?

Efforts will be made to keep your personal information private and confidential. Absolute confidentiality cannot be guaranteed. If information from this study is presented, you will not be identifiable. You will be assigned a coded number. The identifiable information from your recordings will be maintained within Texas A&M College of Dentistry. We will protect your records so that your name and any identifying information will be kept private. The chance that this information will be given out to someone else is very small. Anonymous and coded summarized data might be shared. No identifiers linking you to this study will be included in any sort of report that might be published or presented without your explicit permission. A description of this clinical trial will be made available on http://www.ClinicalTrials.gov. This Web site will not include information that can identify you. At most, the Web site will include a summary of the results. You can search this Web site at any time. This consent form will be filed securely in an official area. People who have access to your information include the Principal Investigator and research study personnel. Representatives of regulatory agencies such as the United States Office of Human Research Protections (OHRP) or the Food and Drug Administration (FDA) and entities such as the Texas A&M University Human Research Protection Program (HRPP) may access your records to make sure the study is being run correctly and that information is collected properly. If there are any reports about this study, your name or other identifiable information will not be in them. Information about you and related to this study will be kept confidential to the extent permitted or required by applicable state and federal laws.

Cost/Compensation:

Costs that you may incur by participating in this study are travel to and parking at the clinic, as well as your time. You or your insurance will be responsible for routine care procedures that includes radiographic images and the periodontal oral examination, which is approximately $281.

You will not be billed for any procedures that are for research purposes only. If you are unsure of your financial responsibility, please discuss this with your insurance or your provider.

Also, you will not be paid for your participation in this study.

The dentist-fitted oral appliance is yours to keep upon completion of the study, also at no cost to you. The approximate value of the periodontal care, radiographs and therapy is $300, and the oral appliance therapy is

IRB NUMBER: IRB2022-1450-CD-FB
IRB APPROVAL DATE: 03/02/2023

$350. In private practice these same therapies could exceed $4500 in total. All participants are required to promptly return the home sleep study kits required by the research coordinator.

In summary, at no cost to you, you will receive over the course of 12 weeks

1. tests to evaluate your sleep quality and breathing during sleep,
2. examinations of your periodontal health including radiographs (x-ray images)
3. comprehensive periodontal therapy (scaling of all four quadrants of your mouth to improve your periodontal health); may also include root planing if needed,
4. oral appliance therapy with mouth-shield (OA+) that may improve your snoring, sleep, and
5. coordinated care from experts in dental sleep medicine and periodontics (a dental specialty).

What if New Findings Occur During the Study?

During the course of the study, you will be informed of any significant new findings (either good or bad), such as changes in the risks or benefits resulting from participation in the research or new alternatives to participation, that might cause you to change your mind about continuing in the study.

If new information is provided to you, your consent to continue participating will be re-obtained.

What Else do I Need to Know?

Clinical care, discomforts and injuries related to this study will be carefully monitored and managed by the dentists on the research team. If you become ill or get injured as a result of the study devices or procedures, you should seek medical treatment through your doctor or treatment center of choice. You should promptly tell the study doctor about any illness or injury.

Texas A&M University has no program to pay for medical care for research-related injury. This does not keep you from seeking to be paid back for care required because of a bad outcome.

Investigator Payment:

The investigators conducting this study are compensated only for their time to do the study and will not get any payment for specific results of the study.

Participant Rights:

Your participation in this research study is voluntary. You may choose not to join or may leave the study at any time. If you choose not to be in this study or stop being in the study, there will be no effect on your dental or medical care, employment, evaluation, student status, academic standing, or relationship with Texas A&M University. This decision will also not involve penalties or loss of benefits to which you are otherwise entitled.

If the investigator decides that participating in this study is not in your best interest (for instance, if you become ill), your participation in the study will be stopped. The principal investigator also has the option to terminate the participation of subjects who are uncooperative or otherwise affect the integrity or conduct of the research. Participation in this study does not guarantee that you will be able to obtain care at Texas A&M University College of Dentistry beyond the scope of this study. After the study, if you wish to become a regular patient of the College, you will need to go through the standard screening process to determine eligibility. This will involve additional cost to you.

IRB NUMBER: IRB2022-1450-CD-FB
IRB APPROVAL DATE: 03/02/2023

Your personally identifiable health information, including your contact information will be retained, but held confidential, for up to 20 years after the completion of the study, unless you opt for the usual retention schedule of 6 years. The 20-year retention period will enable the researchers to evaluate long-term responses to oral appliance therapy, which has not been done before; it will enable the research team to invite you back to the clinic for regular recall appointments (for example, every 6 months). Only non-invasive oral exams and sleep studies and data collection, such as done in the initial study, would be taken. Please check one of the following boxes:

I agree to allow the investigators to retain my personally identifiable data for ☐ 6 or ☐ 20 years.

For questions about this research study contact: Emet Schneiderman, PhD emet@tamu.edu, (214)-828-8377 or the Clinical Research Coordinator at 214-828-8291. If you experience a research-related injury that is not serious, call (214)-828-8291; if serious or an emergency, call 911. For questions about your rights as a research participant or if you have questions, complaints, or concerns about the research, you may call the Texas A&M University Human Research Protection Program office at (979) 458-4067 or send an email to the office: irb@tamu.edu.

I agree to participate in this study. I have read all the above or have heard it read to me. I have had the opportunity to ask questions about this study, and my questions have been answered to my satisfaction. I consent to release my records to the research staff.

1. I clearly understand that this is a research study.
2. I clearly understand the risks associated with participation in this study.
3. I clearly understand the length of time during which I will be participating in this study.
4. I clearly understand the purpose and anticipated outcomes of this study.
5. I clearly understand that my participation in this study is voluntary.
6. I clearly understand that my participation in this study does not affect my legal rights.
7. I certify that I am 18 years of age or older.

Signature Block for Capable Adult

____ _______________________

Signature of subject Date

____

Printed name of subject

____ _______________________

Signature of person obtaining consent Date

____

Printed name of person obtaining consent

YOU WILL BE GIVEN A SIGNED COPY OF THIS CONSENT DOCUMENT TO KEEP

IRB NUMBER: IRB2022-1450-CD-FB
IRB APPROVAL DATE: 03/02/2023
