# Supplementary material for: Effects of Oral Appliance Therapy with a Mouth Shield in Periodontitis Patients Who Snore: A Split-Mouth Randomized Controlled Trial
Source: Dent J (Basel). 2025 Jun 27;13(7):292. doi: 10.3390/dj13070292 (PMC12294119; doi:10.3390/dj13070292)
Supplement: Supplementary file 1 [file dentistry-13-00292-s001.zip › dentistry-3580690-supplementary/dentistry-3580690-supplementary/File 3. OA+ Perio Inclusion criteria 11-22-22 4-17-25.pdf]

Study Population Characteristics

Conditions or Focus of Study:

Adults with mild to moderate periodontal disease and sleep disturbed breathing

Inclusion Criteria:

1. Mouth breathing and snoring confirmed by home sleep test (NOX T3; ≥4 snores/hour) and complaints of excessive daytime sleepiness
2. Seeking treatment for periodontitis (stage 1 or 2 as confirmed by full periodontal examination at Visit 1 or 2)
3. Adults 18-85 years old
4. Stable medical condition (e.g., diabetes or hypertension properly managed)
5. At least 8 natural maxillary teeth to support the oral appliance
6. Mallampati score from I to III; Palatine tonsils – grade 0, 1, or 2
7. Central and mixed apnea index < 5 events/hour
8. Able to speak, read, and comprehend English fluently
9. At least 12 years of education
10. Ability to apply and remove home sleep recorder

Willingness to

11. Share PHI, medication list, current and past medical and dental information with research team members as needed
12. Follow all oral hygiene instructions and attend all appointments (periodontal and otherwise) as stipulated by the protocol
13. Allow the collection of plaque and waste materials from periodontal procedures and allow them to be evaluated for bacterial DNA (not human DNA)
14. Provide saliva samples (spit into tube)
15. Wear the oral appliance (OA) nightly as instructed for 12 weeks and with the mouth-shield (OA+) for the last 8 weeks
16. Complete brief surveys on sleep and related subjective experiences
17. Communicate with the clinical research coordinator regarding OA titration
18. Continue current medication and supplement use
19. Wait till the end of 12-week experimental period to complete periodontal therapy; this will include scaling, root planing of untreated side of dentition as well as redoing the treated side

Exclusion Criteria

1. Gingival probing depths > 5mm
2. Tooth mobility score greater than 2
3. Loose crowns or fillings.
4. Removable dentures or bridges; Temporary crowns; orthodontic braces.
5. Severe xerostomia
6. Severe medical illness such as symptomatic chronic obstructive pulmonary disease, renal failure, symptomatic coronary or cerebral vascular disease, cardiac dysrhythmia (i.e., atrial fibrillation); pacemaker; cardiopulmonary dysfunction (i.e., chronic heart failure),

7. Severe psychiatric and neurological disorders such as current substance abuse (including alcohol, nicotine); major depression and psychotic disorder; seizure disorder
8. Morbid obesity (Body Mass Index: BMI $\geq$35)
9. Active temporomandibular joint disorder (TMD) or jaw muscle pain; persistent history of TMD
10. Diagnosis of severe periodontitis
11. Morphological airway abnormalities (malformations of throat; e.g., a very small airway due to a condition such as severe micrognathia or Pierre Robin Syndrome)
12. Restrictions in jaw opening (difficult opening mouth widely) < 30 mm
13. Pre-existing difficulty swallowing; throat or neck related health issues;
14. Previous major surgery to throat and surrounding area such as UPPP (uvulopalatopharyngoplasty)
15. Intellectual disability that would prevent giving informed consent
16. Serious hormonal disease (endocrine dysfunction such as Addison's disease, Cushing's disease, hyperthyroidism); Diabetes and osteoporosis are OK)
17. Pregnant, breast feeding or intent to become pregnant during the study
